# EPCAM and TROP2 share a role in claudin stabilization and development of intestinal and extraintestinal epithelia in mice

**DOI:** 10.1242/bio.059403

**Published:** 2022-07-11

**Authors:** Roman Szabo, Jerrold M. Ward, Ferruh Artunc, Thomas H. Bugge

**Affiliations:** 1 Proteases and Tissue Remodeling Section, National Institute of Dental and Craniofacial Research, National Institutes of Health, Bethesda, MD 20892, USA; 2 Global Vet Pathology, Montgomery Village, MD 20886, USA; 3 Department of Internal Medicine, Division of Endocrinology, Diabetology and Nephrology, University Hospital Tübingen, 72076 Tübingen, Germany; 4 Institute of Diabetes Research and Metabolic Diseases (IDM) of the Helmholtz Center Munich at the University Tübingen, 72076 Tübingen, Germany; 5 German Center for Diabetes Research (DZD) at the University Tübingen, 72076 Tübingen, Germany

**Keywords:** Claudin stabilization, Enteropathy, Epithelial development, Hyperkeratosis, Proteinuria

## Abstract

Epithelial cell adhesion molecule (EPCAM) is a transmembrane glycoprotein expressed on the surface of most epithelial and epithelium-derived tumor cells and reported to regulate stability of epithelial tight junction proteins, claudins. Despite its widespread expression, loss of EPCAM function has so far only been reported to prominently affect intestinal development, resulting in severe early onset enteropathy associated with impaired growth and decreased survival in both humans and mice. In this study, we show that the critical role of EPCAM is not limited to intestinal tissues and that it shares its essential function with its only known homolog, Trophoblast cell surface antigen 2 (TROP2). EPCAM-deficient mice show significant growth retardation and die within 4 weeks after birth. In addition to changes in small and large intestines, loss of EPCAM results in hyperkeratosis in the skin and forestomach, hair follicle atrophy leading to alopecia, nephron hypoplasia in the kidney, proteinuria, and altered production of digestive enzymes by the pancreas. Expression of TROP2 partially, but not completely, overlaps with EPCAM in a number developing epithelia. Although loss of TROP2 had no gross impact on mouse development and survival, TROP2 deficiency generally compounded developmental defects observed in EPCAM-deficient mice, led to an approximately 60% decrease in embryonic viability, and further shortened postnatal lifespan of born pups. Importantly, TROP2 was able to compensate for the loss of EPCAM in stabilizing claudin-7 expression and cell membrane localization in tissues that co-express both proteins. These findings identify overlapping functions of EPCAM and TROP2 as regulators of epithelial development in both intestinal and extraintestinal tissues.

## Abstract (precis)

**Summary:** Closely related paralogs EPCAM and TROP2 have distinct but overlapping functions in claudin stabilization and mouse postnatal development.

## INTRODUCTION

Epithelia are essential for proper development and homeostasis of organs by creating a barrier that protects tissues from external physical, chemical and microbial insults and limits unwanted diffusion of molecules (Marchiando et al., 2010). Functionality of epithelia depends on the ability of individual cells to form stable contacts with their neighbors and the underlying extracellular matrix. These epithelial cell­­–cell and cell–matrix adhesions are critical regulators of cell polarity, migration, proliferation, and differentiation, and are largely mediated by several classes of cell adhesion molecules (CAMs) that include cadherins and integrins (Marchiando et al., 2010; Garcia et al., 2018).

EPCAM (also known as Trop1 or CD326) is a type I transmembrane glycoprotein that was originally identified as a novel tumor-specific antigen highly expressed on the surface of many carcinoma cells (Herlyn et al., 1979). High EPCAM expression is often associated with poor prognosis and is linked to tumor cell proliferation and metastasis (Spizzo et al., 2002; Varga et al., 2004; Brunner et al., 2008; Laimer et al., 2008; Chen et al., 2014). In addition to tumors, EPCAM is also present on the basolateral surface of most developing and adult epithelia, and during tissue regeneration, where active proliferation typically correlates with high expression and differentiation with a significant downregulation of the protein (Klein et al., 1987; Schiechl and Dohr, 1987; Balzar et al., 1999; Schmelzer and Reid, 2008; Trzpis et al., 2008).

Despite its name, EPCAM does not fit into any of the ‘classical’ CAM families and its role in cell adhesion remains unclear. Ectopic expression of EPCAM stimulates Ca2+-independent cell aggregation and formation of intercellular contacts in cells that normally lack cell–cell interactions (Litvinov et al., 1994). However, in cells interconnected by classic cadherins, EPCAM expression had either no impact, or actually weakened intercellular adhesion by interfering with the binding of cadherins to alpha-actinin and actin cytoskeleton, suggesting that EPCAM may suppress, rather than promote, cell adhesion (Litvinov et al., 1997; Gaiser et al., 2012; Tsaktanis et al., 2015). EPCAM has also been proposed to be involved in the formation and stabilization of epithelial tight junctions (TJs) by regulating cellular distribution and degradation of several TJ proteins, such as claudin-7 and claudin-1 (Ladwein et al., 2005; Lei et al., 2012; Wu et al., 2013, 2017). This is consistent with a loss of expression of selected claudins in humans and mice with EPCAM mutations, and strong similarities in phenotypes of mice lacking EPCAM and claudin-7 (Ding et al., 2012; Lei et al., 2012; Mueller et al., 2014; Tanaka et al., 2015).

Although the precise molecular function of EPCAM is not yet fully understood, its critical role in development is quite well established. In humans, loss-of-function mutations in *EPCAM* gene lead to congenital tufting enteropathy (CTE), a rare and severe form of early-onset enteropathy associated with chronic diarrhea and impaired growth (Sivagnanam et al., 2008). CTE is characterized by intestinal epithelial dysplasia, villous atrophy and formation of distinctive ‘tufts’ in the small intestinal and colonic mucosae, leading to intestinal failure and reliance on total parenteral diet (Sherman et al., 2004; Goulet et al., 2007; Mueller et al., 2014). Similarly, mice carrying inactivating mutations in *Epcam* develop severe hemorrhagic enteropathy, inability to gain weight and postnatal lethality within 5-10 days after birth (Guerra et al., 2012; Lei et al., 2012).

The only known homolog of EPCAM, TROP2 (also known as tumor-associated calcium signal transducer 2, TACSTD2), shares 49% sequence identity and 67% sequence similarity with EPCAM, and, like EPCAM, is expressed in a wide range of epithelia (Stepan et al., 2011; Trerotola et al., 2013). Loss of TROP2 function in humans results in gelatinous drop-like corneal dystrophy (GDLD), characterized by accumulation of gelatinous amyloid masses within or beneath corneal epithelium, leading to blindness (Tsujikawa et al., 1999). Similarly, TROP2-deficient mice are fully viable, but display increased susceptibility to corneal opacity later in life (Wang et al., 2011; Nagahara et al., 2020).

Given the prominent and widespread expression of EPCAM and TROP2 in epithelial tissues, the highly tissue-restricted impact of their respective loss of function mutations is rather surprising. With the exception of biliary ductopenia observed in some CTE patients, humans or mice lacking EPCAM were not reported to exhibit any prominent extraintestinal phenotypes, whereas the effect of TROP2 inactivation appears to be limited to corneal epithelium (Tsujikawa et al., 1999; Sivagnanam et al., 2008; Guerra et al., 2012; Lei et al., 2012; Zhan et al., 2021). It is plausible that the lack of more widespread phenotype may result from a functional redundancy between the two proteins in developing and adult epithelia (Balzar et al., 1999; Nakatsukasa et al., 2010). Consistent with this notion, TROP2 was also found to bind to claudin-7, and a combined knockdown of both EPCAM and TROP2 was needed to dramatically decrease claudin levels in cultured human keratinocytes (Takaoka et al., 2007; Nakatsukasa et al., 2010; Wu et al., 2020). Indeed, it has been suggested that lack of TROP2 expression in intestinal epithelium may explain the specific vulnerability of this tissue to the loss of EPCAM, as ectopic expression of TROP2 in intestinal epithelial cells suppressed CTE-like defects in EPCAM-deficient mice (Nakatsukasa et al., 2010; Nakato et al., 2020). Furthermore, a loss of function of *tacstd*, which is the only *EPCAM*-like gene in zebrafish, does result in defects in a variety of tissues and a loss of viability early in development (Slanchev et al., 2009; Lu et al., 2013).

In this study, we show that EPCAM and TROP2 exhibit a widespread but only partially overlapping expression in developing epithelia. Whereas loss of TROP2 alone had no obvious impact on mouse development and survival, EPCAM deficiency affected development of several major organs besides the intestines, including the skin, kidneys, stomach and pancreas, and an additional loss of TROP2 generally compounded developmental defects observed in EPCAM-deficient mice. Our data reveal that the critical role of EPCAM is not limited to intestinal tissues and that EPCAM and TROP2 have overlapping functions in epithelial development.

## RESULTS

### EPCAM and TROP2 have overlapping but distinct expression in mouse epithelia

**Fig. 1.**
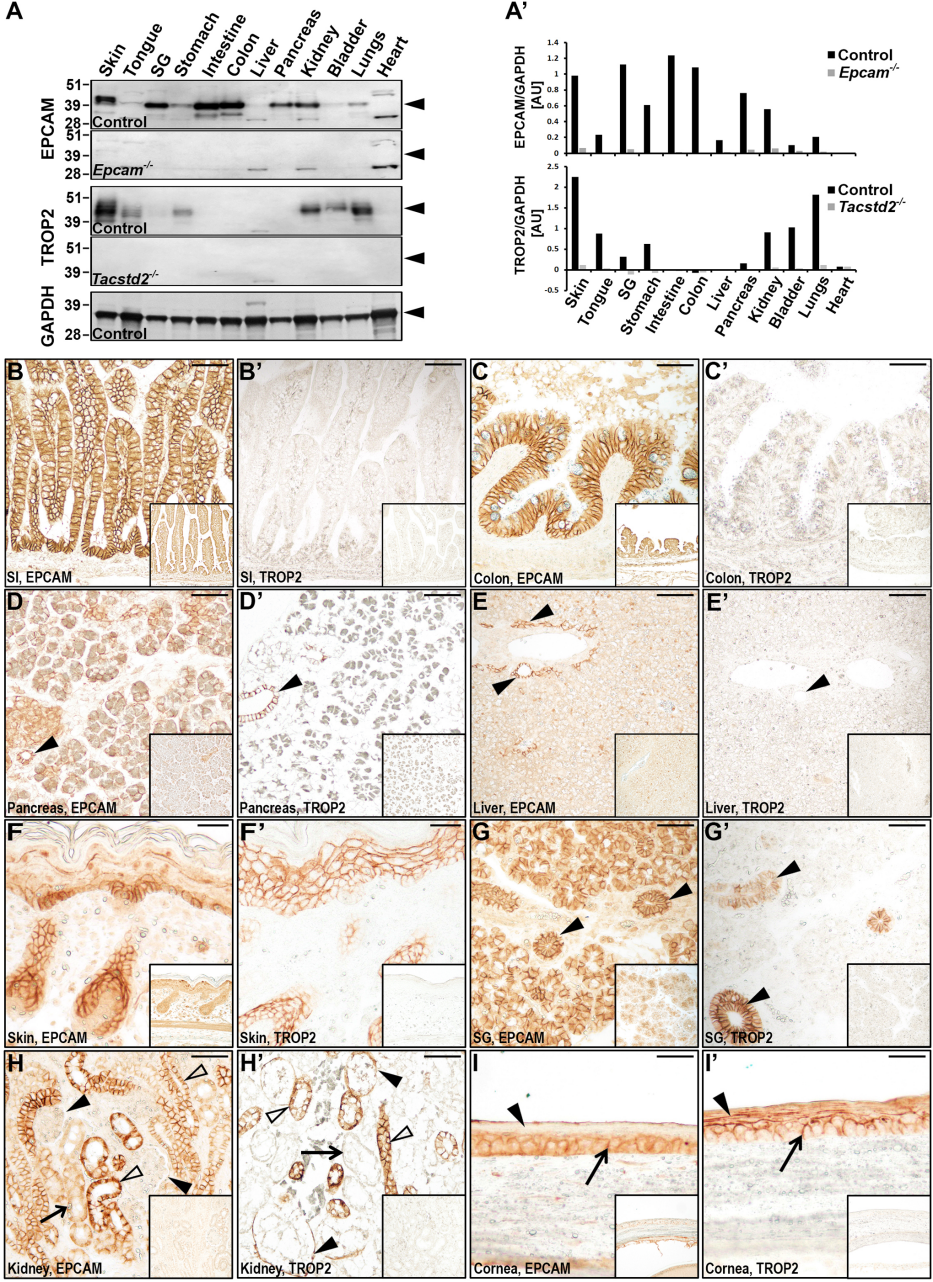
**EPCAM and TROP2 are co-expressed in developing mouse epithelia.** (A) Western blot detection of EPCAM (top two panels) and TROP2 (middle two panels) proteins in tissues from 14 days old wildtype (top and middle, Control), *Epcam^−/−^* (top, lower panel) and *Tacstd2^−/−^* (middle, lower panel) mice. Expression of GAPDH (bottom) was used as loading control for wildtype samples. Predicted position of the protein signal is indicated on the right (arrowheads). Positions of molecular weight markers (kDa) are indicated at the left. EPCAM and TROP2 exhibit widespread and partially overlapping expression. (A′). Quantification of Western blot signals for EPCAM (top) and TROP2 (bottom) proteins, relative to GAPDH. (B-I′). Immunohistochemical analysis of EPCAM and TROP2 expression in the small intestine (SI), colon, pancreas, liver, skin, and salivary gland (SG), and kidney from newborn, and cornea from 2-week-old mice. Tissues from corresponding EPCAM-deficient or TROP2-deficient mice were used as antibody specificity controls (insets). Pancreatic (D,D′), bile (E,E′), and salivary (G,G′) ducts, parietal epithelium of Bowman's capsule (H, H′), and differentiated keratinocytes in cornea (I,I′) are indicated by arrowheads. Proximal tubules in kidney (H,H′) and basal keratinocytes in cornea (I,I′) are indicated by arrows. Distal tubules and collecting ducts in kidney are indicated by open arrowheads (H,H′). Scale bars: B,B′,E, E′, 75 μm; C-D′,F-H′, 50 μm; I,I′, 25 μm.

Despite the reported broad expression of EPCAM and TROP2 in mouse and human tissues, the extent to which the two proteins are co-expressed within the various epithelia is not well established (Momburg et al., 1987; Stepan et al., 2011). As specific tissue localization is likely to determine any potential functional interaction, we first set out to perform a direct comparison of tissue and cell distribution of EPCAM and TROP2 in developing mice. Western blot analysis of major epithelia-containing organs revealed widespread but distinct expression of the two proteins. Bands corresponding to full length EPCAM and multiple differentially glycosylated forms of TROP2 (Stepan et al., 2011) were detected in most organs, including skin, tongue, stomach, kidney, bladder, and lungs (Fig. 1A,A′). While no tissue in this panel exclusively expressed TROP2, several appeared to only express EPCAM, including the small and large intestines as well as pancreas. Neither of the proteins was conclusively detected in the liver or heart, consistent with their low content or lack of typical epithelia (Fig. 1A,A′).

Immunohistological analysis confirmed a widespread, membrane-associated, and distinctly epithelium-specific expression of both EPCAM and TROP2 across the neonatal tissues of the gastrointestinal, respiratory, urinary, and integumentary systems (Fig. 1B-I′; Fig. S1). As previously reported, no TROP2 expression was detected in either small or large intestines, in contrast to a prominent expression of EPCAM in both of these tissues (Fig. 1B-C′). Consistent with the result of our Western blot analysis, EPCAM was widely expressed in the epithelia of endo- and exocrine pancreas, whereas expression of TROP2 in this organ was restricted to ductal epithelium (Fig. 1D,D′, arrowheads). EPCAM, but not TROP2, was also detected in the bile duct epithelium in liver (Fig. 1E,E′, arrowheads). In addition, several of the organs that expressed both EPCAM and TROP2 showed only partially overlapping spatial distribution of the two proteins. In skin, EPCAM is largely restricted to developing hair follicles and the basal keratinocytes of the adjacent interfollicular epidermis, whereas TROP2 is detected in both the hair follicles and throughout the epidermis, including the more differentiated keratinocytes of spinous and granular layers (Fig. 1F,F′). In salivary glands, both proteins were detected in epithelial cells of main striated and excretory salivary ducts, in contrast to the acinar cells that only express EPCAM (Fig. 1G,G′, arrowheads). In developing kidneys, EPCAM is widely expressed along the ductal epithelia of the nephron, whereas expression of TROP2 appears to be more restricted to distal tubules and collecting ducts (Fig. 1H,H′, open arrowheads) but is also detected in parietal epithelium of the Bowman's capsule surrounding glomeruli (Fig. 1H,H′, arrowheads). Both proteins were also detected in the corneal epithelium, although similar to epidermis, expression of EPCAM appears to be restricted to basal keratinocytes (Fig. 1I,I′, arrows), whereas TROP2 is present in both basal and differentiated layers (Fig. 1I,I′, arrows and arrowheads, respectively).

On the other hand, EPCAM and TROP2 do co-express in a significant subset of tissues that includes epithelia of oral and nasal cavities, thymus, keratinized epithelium of forestomach, as well as bronchiolar and alveolar epithelia in lungs (Fig. S1, arrowheads). Apparent strong expression of EPCAM in the glandular stomach was also detected in control tissues from EPCAM-deficient mice and, therefore, could not be evaluated (Fig. S1F,F′). In summary, these findings document both overlapping and distinct expression of EPCAM and TROP2 in developing epithelia.

### Individual loss of EPCAM or TROP2 does not affect mouse embryonic survival

Despite the wide tissue distribution of the two proteins in developing epithelia, TROP2 has been reported to be dispensable for mouse development and survival under normal housing conditions, whereas the effect of EPCAM deficiency has been reported to be largely confined to the intestines (Wang et al., 2011; Guerra et al., 2012; Lei et al., 2012). This unexpected lack of a more widespread phenotype has been ascribed to a possible functional compensation between the two proteins (Nakato et al., 2020). To test this hypothesis, especially in light of the rather limited co-expression of EPCAM and TROP2 in several of epithelia (see above), we performed a systematic phenotypic analysis of mice with single or combined EPCAM and TROP2 deficiency. To generate single-deficient mice, two CRISPR guide RNAs per gene were designed to target exon 4 of mouse *Epcam* gene and a 5′ region in the coding sequence of the intronless *Tacstd2* gene encoding TROP2 (Fig. S2A, Table S1). Out of several founder mice with identified inactivating mutations, lines that carry 20 bp and 61 bp deletions corresponding to, respectively, nt.261-280 of *Epcam* and nt.57-117 of *Tacstd2* open reading frames were selected for further analysis (Fig. S2A,B). Both mutations result in a frameshift and a premature termination of translation (p.I88VfsX53 for EPCAM and p.R20QfsX24 for TROP2) and are expected to be null. Indeed, Western blot and immunohistochemical analysis confirmed complete lack of detectable EPCAM or TROP2 proteins in postnatal tissues from their corresponding knockout lines (Fig. 1A, and insets in Fig. 1B-I′).

**Fig. 2.**
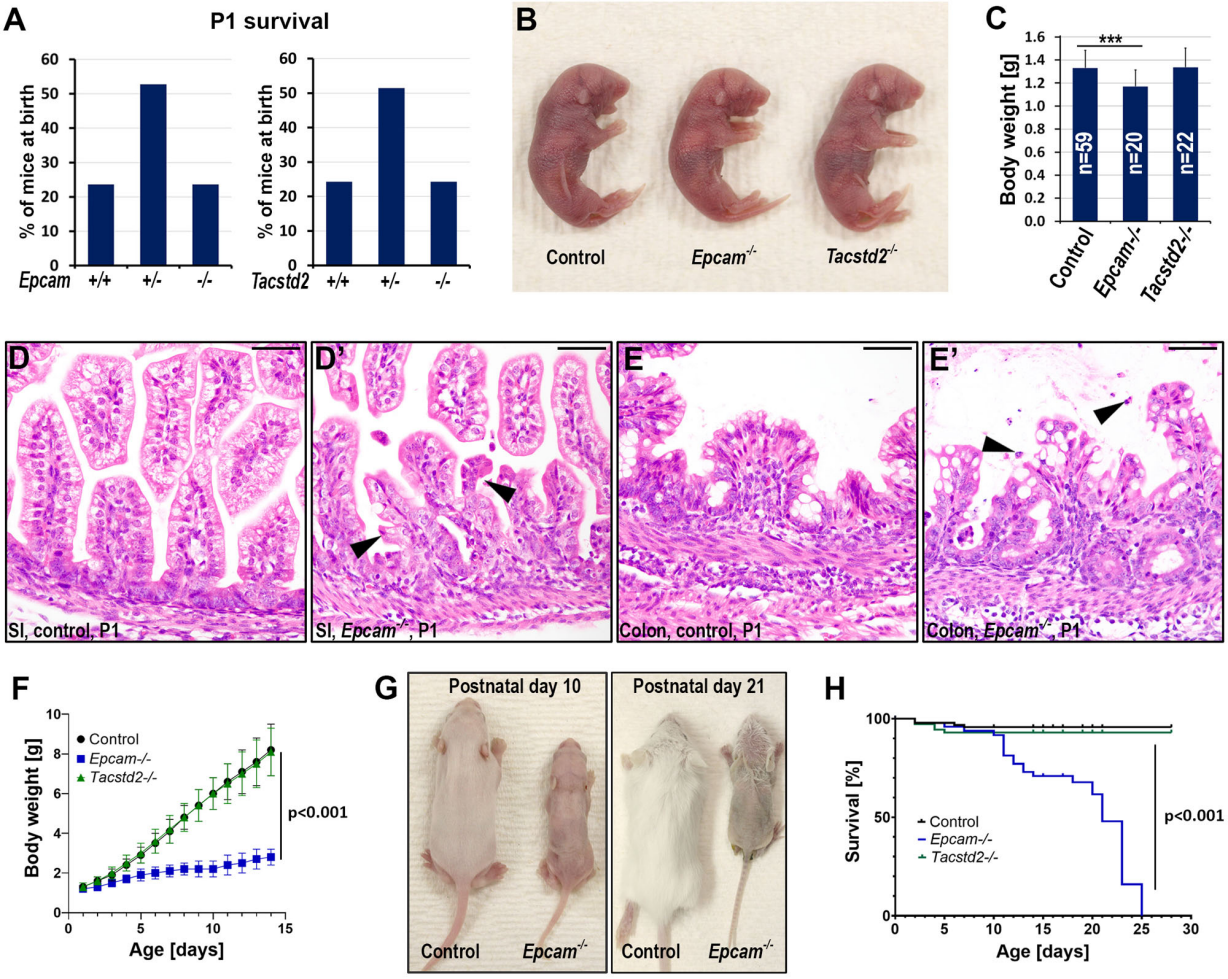
**Genetic inactivation of the *Epcam* or *Tacstd2* genes does not affect mouse embryonic survival.** (A) Distribution of genotypes among newborn offspring from *Epcam^+/−^×Epcam^+/−^* (n=72, left), and *Tacstd2^+/−^×Tacstd2^+/−^* (*n*=70, right) breeding pairs. All genotypes were presented in the expected Mendelian ratios. (B,C) Outward appearance (B) and body weight (C) of control (left), *Epcam^−/−^* (middle), and *Tacstd2^−/−^* (right) newborn mice. *P*-value: ***<0.001 (*Epcam^−/−^* versus control, two-tailed Student's *t*-test). (D-E′) H&E stain of small intestines (SI) and colons from newborn (P1) control and their littermate *Epcam^−/−^* mice. Examples of epithelial tufting (D′) and shedding of cells (E′) in *Epcam^−/−^* tissues are indicated by arrowheads. (F) Postnatal gain in body weight of control (black), *Epcam^−/−^* (blue), and *Tacstd2^−/−^* (green) mice. (G) Outward appearance of 10- and 21-day-old control mice (left) and their *Epcam^−/−^* littermates (right). EPCAM-deficient mice present with diminished growth and near-complete lack of pelage hair. (H) Postnatal survival of control (black), *Epcam^−/−^* (blue) and *Tacstd2^−/−^* (green) mice. All EPCAM-deficient mice die before 4 weeks of age with median survival of 23 days. *P*-value <0.001, *Epcam^−/−^* versus control, Log-rank test (Mantel-Cox). Scale bars: D-E′, 50 μm.

Analysis of the allele distribution among the newborn offspring from *Epcam* and *Tacstd2* heterozygous breeding pairs revealed expected numbers of the corresponding single-deficient mice, suggesting that the absence of either of the two proteins is fully compatible with mouse embryonic survival (Fig. 2A). None of the single-deficient mice exhibited any outward developmental abnormalities at birth, except for a slightly lower body weight observed in EPCAM-deficient mice (Fig. 2B,C). As expected, histological examination of newborn tissues from EPCAM-deficient mice revealed abnormalities in the appearance of intestinal tissues, including villous atrophy and epithelial tufting in small intestine and epithelial erosion in colon (Fig. 2D-E′, arrowheads). No extra-intestinal macroscopic or histological abnormalities were observed in EPCAM knockouts at this age. Similarly, no changes were apparent in any of the organs from newborn TROP2-deficient mice.

### EPCAM is critical for postnatal development of both intestinal and extraintestinal tissues

Postnatally, EPCAM knockouts show minimal weight gain and can be easily distinguished from their control littermates by their smaller size by the end of the first week and a near-complete lack of pelage hair thereafter (Fig. 2F,G). All EPCAM-deficient mice die before reaching 4 weeks of age with a median survival of 23 days (Fig. 2H). In contrast, no obvious growth defects or a loss of viability were observed in mice lacking TROP2 (Fig. 2F,H).

**Fig. 3.**
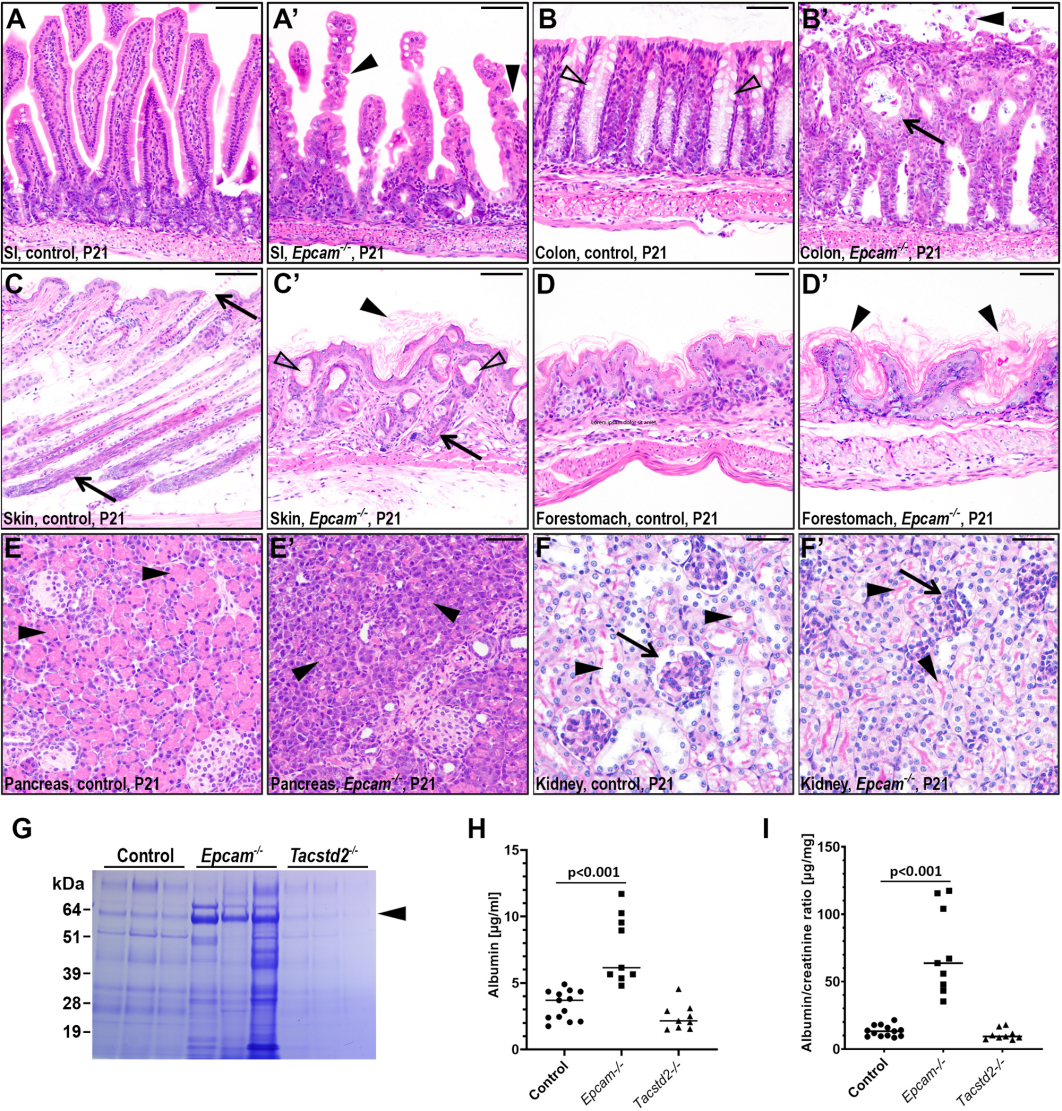
**Loss of EPCAM leads to widespread defects in postnatal epithelial development.** (A) Postnatal gain in body weight of control (black), *Epcam^−/−^* (blue), and *Tacstd2^−/−^* (green) mice. (B,C) Outward appearance of 10- (B) and 21- (C) day-old control mice (left) and their *Epcam^−/−^* littermates (right). EPCAM-deficient mice present with diminished growth and near-complete lack of pelage hair. (D) Postnatal survival of control (black), *Epcam^−/−^* (blue), and *Tacstd2^−/−^* (green) mice. All EPCAM-deficient mice die before 4 weeks of age with median survival of 23 days. *P*-value <0.001, *Epcam^−/−^* versus control, Log-rank test (Mantel-Cox). (E-J′) H&E (E-H′, J, J′) and periodic acid Schiff (PAS) (I, I′) stain (A-F′) H&E stain of small intestine (SI), colon, skin, forestomach and pancreas, and periodic acid Schiff (PAS) stain of kidney (F,F′) from 21-day-old control (E-J) and their littermate *Epcam^−/−^* (E'-J′) mice. Loss of EPCAM resulted in villous atrophy and tufting (A′, arrowheads) in small intestines, loss of mucin-producing goblet cells (B, open arrowheads), epithelial erosion (B′, arrowheads) and cyst formation (B′, arrows) in colon, poorly developed hair follicles and an atrophy of hair shafts (C,C′, arrows), keratin-filled utricles (C′, open arrowheads), and hyperkeratosis in skin and forestomach (C′ and D′, arrowheads), markedly decreased volume of secretory granules in exocrine pancreas (E,E′, arrowheads), and reduced tubular lumen (F,F′, arrowheads) and Bowman's space (F,F′, arrows) in kidneys. (G-I K-M). Representative Coomassie stain (GK), albumin concentration (HL) and albumin-to-creatinine ratio (IM) in urine extracted from 14-21-day-old control, *Epcam^−/−^* and *Tacstd2^−/−^* mice. EPCAM-deficient mice exhibit signs of severe proteinuria. Arrowhead in (GK) indicates predicted position of albumin, and positions of molecular weight markers (kDa) are indicated on the left. Each lane in G and each data point in H and I represent an individual mouse. Scale bars: A-C', 75 μm; D-E′, 50 μm; F,F′, 25 μm.

**Fig. 4.**
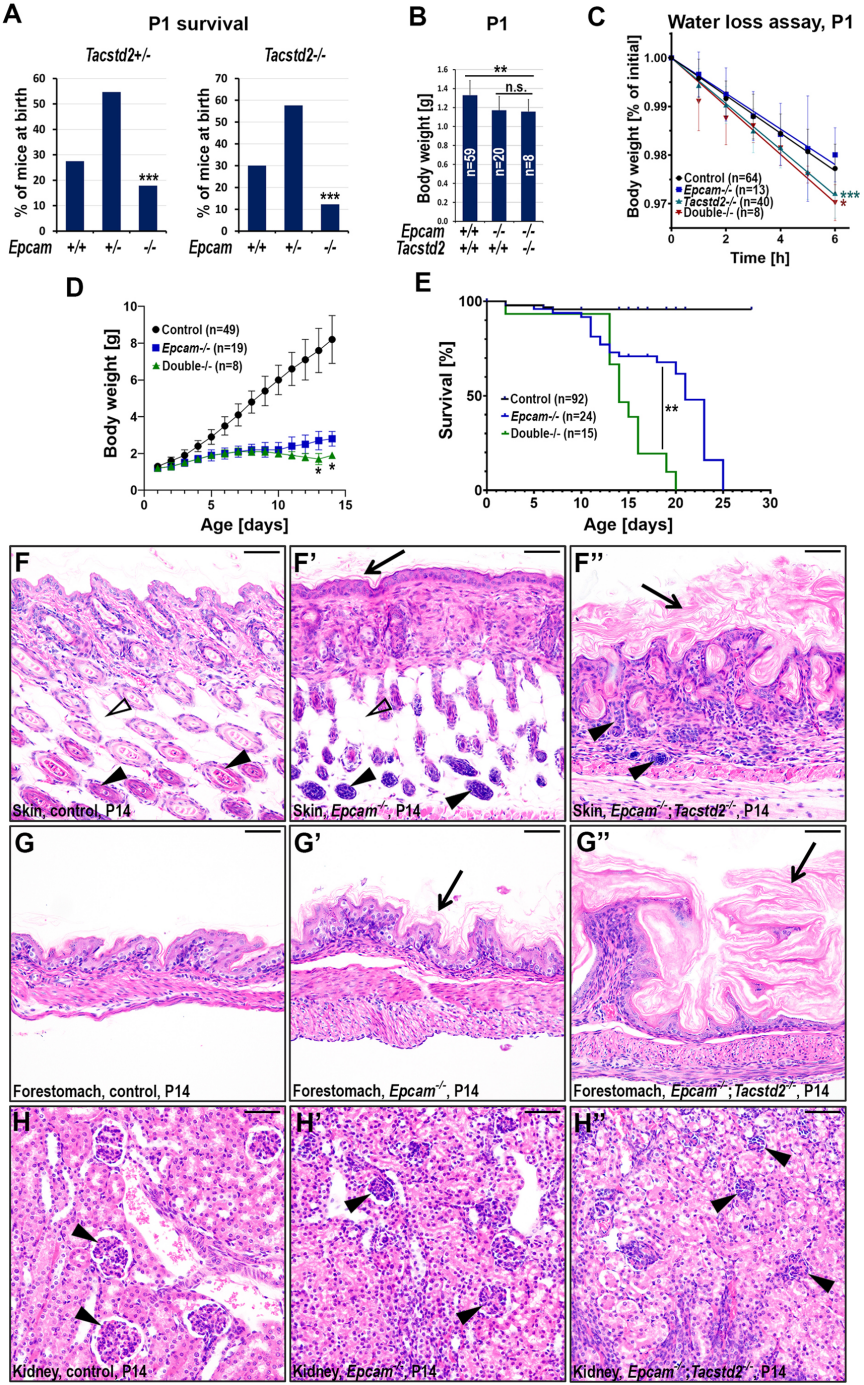
**Loss of TROP2 increases severity of developmental defects in EPCAM-deficient mice.** (A) Distribution of *Epcam* genotypes among *Tacstd2^+/−^* (*n*=269, left) and *Tacstd2^−/−^* (*n*=163, right) newborn (P1) offspring from *Epcam^+/−^*;*Tacstd2^+/−×^Epcam^+/−^*;*Tacstd2^+/−^* breeding pairs. Loss of one or both wildtype alleles of Tacstd2 gene decreased prenatal survival of EPCAM-deficient embryos by 25 and 60%, respectively. *P*-value: ***<0.001 (chi-square test). (B) Total body weight of littermate control (left), *Epcam^−/−^* single-deficient (middle), and *Epcam^−/−^;Tacstd2^−/−^* double-deficient (right) mice at birth (P1). *P*-value: **<0.01 (two-tailed Student's *t*-test). (C) Transepidermal water loss over a 6 h period in newborn (P1) control (black), *Epcam^−/−^* single-deficient (blue), *Tacstd2^−/−^* single-deficient (green)*^−^*and *Epcam^−/−^;Tacstd2^−/−^* double-deficient (double*^−/−^*, red) mice. Increased water loss was detected in mice lacking TROP2 (*Tacstd2^−/−^* and *Epcam^−/−^;Tacstd2^−/−^*). *P*-value: *<0.05, ***<0.001 (compared to control, simple linear regression slope comparison). (D, E) Postnatal gain in body weight (D) and survival (E) in control (black), *Epcam^−/−^* (blue), and *Epcam^−/−^*;*Tacstd2^−/−^* (green) mice. (D) Loss of TROP2 further limited weight gain in *Epcam^−/−^* mice after day 10 and decreased median survival from 23 to 14 days. *P*-value: *<0.05, **<0.01 [*Epcam^−/−^* versus double*^−/−^*, two-tailed Student's *t*-test, (D), Log-rank (Mantel–Cox) (E)]. (F-H″) H&E stain of skin, forestomach, and kidney from a 14-day-old control mouse, and their littermate *Epcam^−/−^* and *Epcam^−/−^;Tacstd2^−/−^* mice. Inactivation of TROP2 in *Epcam-*deficient mice increased severity of hair follicle hypoplasia (F-F″, arrowheads), and loss of subcutaneous fat tissue (F-F″, open arrowheads) in skin, hyperkeratosis in both skin and forestomach (F-G″, arrows), and glomerular hypoplasia in kidney (H-H″, arrowheads), compared to *Epcam^−/−^* single-deficient mice. Scale bars: F-H″, 50 μm.

Consistent with previous reports, loss of EPCAM led to a progressive deterioration of intestinal epithelia, resulting in epithelial tufting, villous dysplasia and general villous atrophy in small intestine (Fig. 3A-A′, arrowheads), and dysplastic development with loss of mucin-producing Goblet cells (Fig. 3B, open arrowheads), cyst formation, and epithelial erosion in the colon (Fig. 3B, arrow and arrowhead, respectively) (Guerra et al., 2012; Lei et al., 2012; Mueller et al., 2014) (Fig. 3A-B′). Both tissues also presented with increased infiltration of CD45-positive immune cells (Fig. S2C, arrowheads). Interestingly, however, EPCAM deficiency also led to previously unreported changes in several other tissues. The lack of pelage hair observed in EPCAM knockouts indicated a defect in hair follicle and/or epidermal development (Fig. 2G). Histological analysis of skin from 3-week-old mice revealed complete absence of well-developed hair follicles and a lack of hair shafts (Fig. 3C,C′, arrows) that were largely replaced by keratin-filled utricles (Fig. 3C′, open arrowheads), as well as moderate hyperkeratosis and acanthosis (Fig. 3C′, arrowheads), and a near-complete lack of subcutaneous adipose tissue. Despite the documented importance of EPCAM in stabilization of claudins and, presumably, epithelial tight junctions, EPCAM-deficient newborn pups did not exhibit any measurable increase in transepidermal water loss, indicating that the histological changes in these mice do not result from an impaired epidermal barrier function (see below, Fig. 4C). Interestingly, hyperkeratosis was also observed in the forestomach, implying that a possible role of EPCAM in the terminal differentiation of keratinized epithelia (Fig. 3D,D′, arrowheads).

Furthermore, Epcam^−/−^ mice exhibited greatly reduced volumes of cytosol within the acinar cells of the exocrine pancreas, suggesting altered production and/or secretion of the secretory zymogen granules containing digestive enzymes (Fig. 3E,E′, arrowheads). This was associated with a substantial increase in the amounts of trypsin, chymotrypsin, and pancreatic elastase detected within these cells, consistent with a defect in trafficking and/or secretion of granules, rather than a decrease in enzyme production (Fig. S3). No apparent changes were detected in the epithelia of endocrine pancreas or pancreatic ducts.

Finally, starting 7-10 days after birth, the kidneys of EPCAM-deficient mice exhibited small glomeruli with greatly diminished capsular (Bowman's) space (Fig. 3F,F′, arrows) and proximal and distal tubules lacking discernable lumen (Fig. 3F,F′, arrowheads). Collecting ducts within the kidney cortex did not appear similarly affected. Analysis of urine from 2-3 week old *Epcam^−/−^* mice revealed severe proteinuria, as documented by a substantially increased protein content, albumin concentration and albumin to creatinine ratio (Fig. 3G-I), demonstrating critical role of EPCAM in kidney development.

Apart from the phenotypes described above, no obvious histological abnormalities were observed in any of the major organs from EPCAM-deficient mice, including the liver, lungs, salivary glands, tongue, thymus, bladder, glandular (non-keratinized) stomach and eye.

### Loss of TROP2 does not impair development and survival but further increases severity of phenotypes in EPCAM knockout mice

In contrast to widespread changes observed in mice lacking EPCAM, loss of TROP2 did not have any perceptible impact on development or survival, and TROP2-deficient mice were indistinguishable in overall appearance, size, or survival from their wildtype littermate controls for up to 12 months of age. Similarly, macroscopic and histological analysis did not reveal any abnormalities in any of the major organs from TROP2 single-knockout (*Epcam+/+;Tacstd2−/−*) mice within the first year of life. However, loss of TROP2 did further aggravate several of the developmental defects seen in EPCAM knockout mice. Thus, whereas individual loss of either of the two proteins did not affect overall embryonic survival, only 75% and 42% of the expected number of EPCAM-deficient mice lacking, respectively, one or both alleles of the *Tacstd2* gene were detected at birth (Fig. 4A, compared with Fig. 2C). Surviving newborn double-deficient mice were smaller than their wildtype littermate controls but not significantly different in size from the EPCAM single-deficient mice (Fig. 4B). They also presented with a small but significant increase in transepidermal water loss, indicating a mild defect in epidermal barrier function (Fig. 4C). Unexpectedly, this barrier defect was also observed in TROP2 single-deficient, but not in EPCAM single-deficient mice, indicating that the epidermal barrier defect stems from the loss of TROP2 rather than EPCAM (Fig. 4C).

Loss of TROP2 further restricted postnatal growth of EPCAM-deficient mice and decreased their median survival from 23 to 14 days (Fig. 4D,E). At 2 weeks of age, the skin of the double-deficient mice presented with near-complete loss of hair follicles and hypodermal adipose tissue, and moderate-to-severe hyperkeratosis and acanthosis, all of which were more prominent than in EPCAM single-deficient littermates (Fig. 4F-F″). Similarly, hyperkeratinization of the epithelium of forestomach and glomerular hypoplasia detected in kidneys of EPCAM-deficient mice appeared to further increase in severity in animals lacking both EPCAM and TROP2 (Fig. 4G-G″, arrows, 4H-H″, arrowheads). In contrast, defects in tissues that do not express TROP2, including the small intestine, colon and pancreas, did not seem to increase in severity in double-deficient mice, and the extent of the damage appeared to be comparable to that seen in EPCAM single-deficient animals (Fig. S4). Similarly, lack of TROP2 did not trigger any obvious macroscopic or histological abnormalities in tissues that were not already affected by loss of EPCAM, including the lungs, liver, salivary glands, glandular stomach, thymus, bladder and eye.

### TROP2 supports expression and cell-surface localization of claudin-7 in the absence of EPCAM

**Fig. 5.**
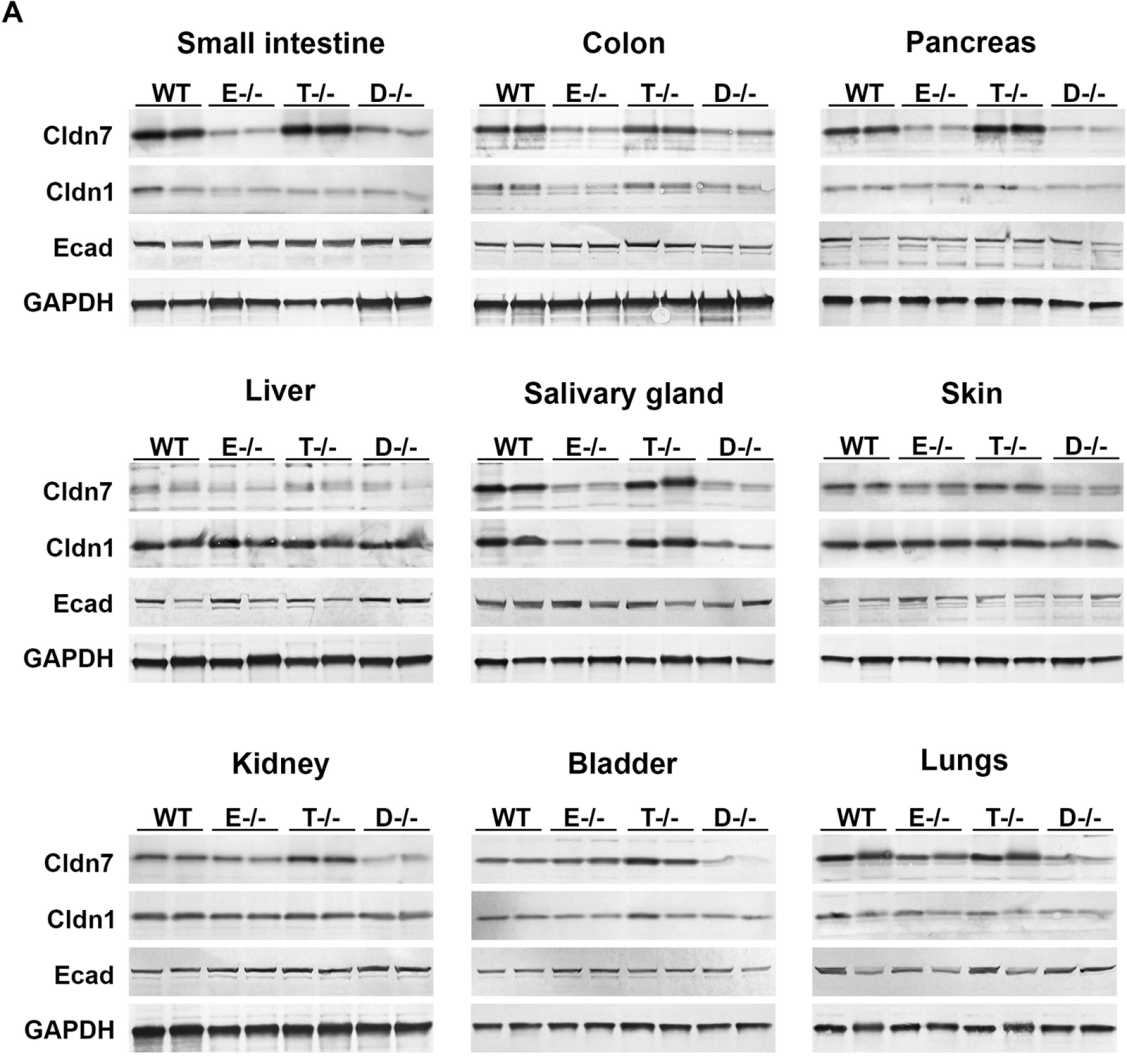
**Expression of epithelial markers in mice lacking EPCAM and/or TROP2.** (A) Western blot detection of claudin-7 (Cldn7), claudin-1 (Cldn1) and E-cadherin (Ecad) in tissues from 14-day-old wildtype (WT, lanes 1 and 2), *Epcam^−/−^* (E*^−/−^*, lanes 3 and 4), *Tacstd^−/−^* (T-/-, lanes 5 and 6) and *Epcam^−/−^;Tacstd2^−/−^* double-deficient (D*^−/−^*, lanes 7 and 8) mice. Expression of GAPDH (bottom) was used as a loading control. TROP2 co-expression helps to stabilize claudin-7 expression in the absence of EPCAM.

EPCAM and TROP2 have previously been reported to assist in stabilization of claudins, with claudin-7 being the primary binding partner (Ladwein et al., 2005; Nakatsukasa et al., 2010; Lei et al., 2012). To address the relative importance of the two proteins for claudin stability and membrane localization in different epithelia, we have analyzed the effect of a single or a combined deficiency in EPCAM and TROP2 on the level and spatial distribution of claudin-7 expression in developing mouse tissues. Western blot analysis of 2-week-old EPCAM-deficient mice further confirmed substantial decrease in levels of claudin-7 in tissues that did not co-express TROP2, such as small and large intestine, pancreas, and liver (Fig. 5, control versus *Epcam^−/−^*). Here, genetic inactivation of TROP2 did not further decrease claudin-7 expression (Fig. 5, Fig. S5, *Epcam^−/−^* versus *Epcam^−/−^;Tacstd2^−/−^*). A similar effect was also observed in salivary gland, where only a small subpopulation of claudin-7-expressing epithelial cells expresses TROP2 (Fig. 5; Figs S5, S1G′). In contrast, tissues where TROP2 is detected in claudin-7-expressing cells, including the skin, kidney, bladder and lungs, loss of EPCAM had either no effect or only partially reduced levels of claudin-7 and a combined EPCAM- and TROP2-deficiency was needed to further suppress claudin-7 expression (Fig. 5; Fig. S5). In addition to claudin-7, levels of another epithelial tight junction marker and a potential EPCAM- and TROP2-binding partner claudin-1 were also reduced in colons and salivary glands from EPCAM-deficient mice, whereas expression of adherens junction marker E-cadherin was not affected (Fig. 5, Fig. S5).

**Fig. 6.**
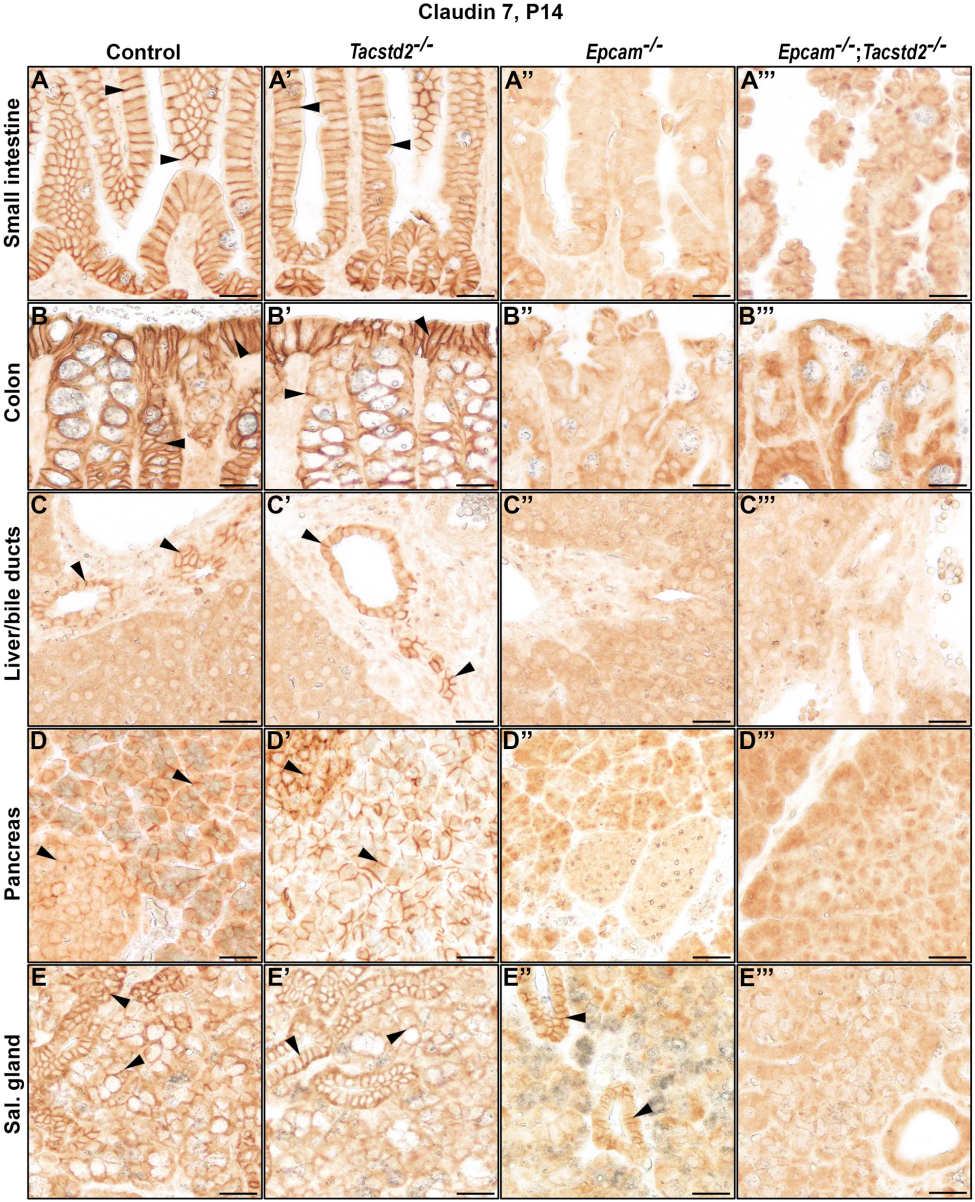
**Cell surface localization of claudin-7 depends on EPCAM and TROP2.** (A-E‴) Immunohistochemical analysis of claudin-7 protein expression in small intestine, colon, liver, pancreas, and salivary gland from 2-week-old control (A-E), *Tacstd2^−/−^* (A′-E′), *Epcam^−/−^* (A″-E″) and *Epcam^−/−^;Tacstd2^−/−^* (A‴-E‴) mice. Cell surface localization of claudin-7 is indicated by arrowheads. Expression of EPCAM or TROP2 is needed to express and localize claudin-7 to the cell surface. Scale bars: 25 μm.

**Fig. 7.**
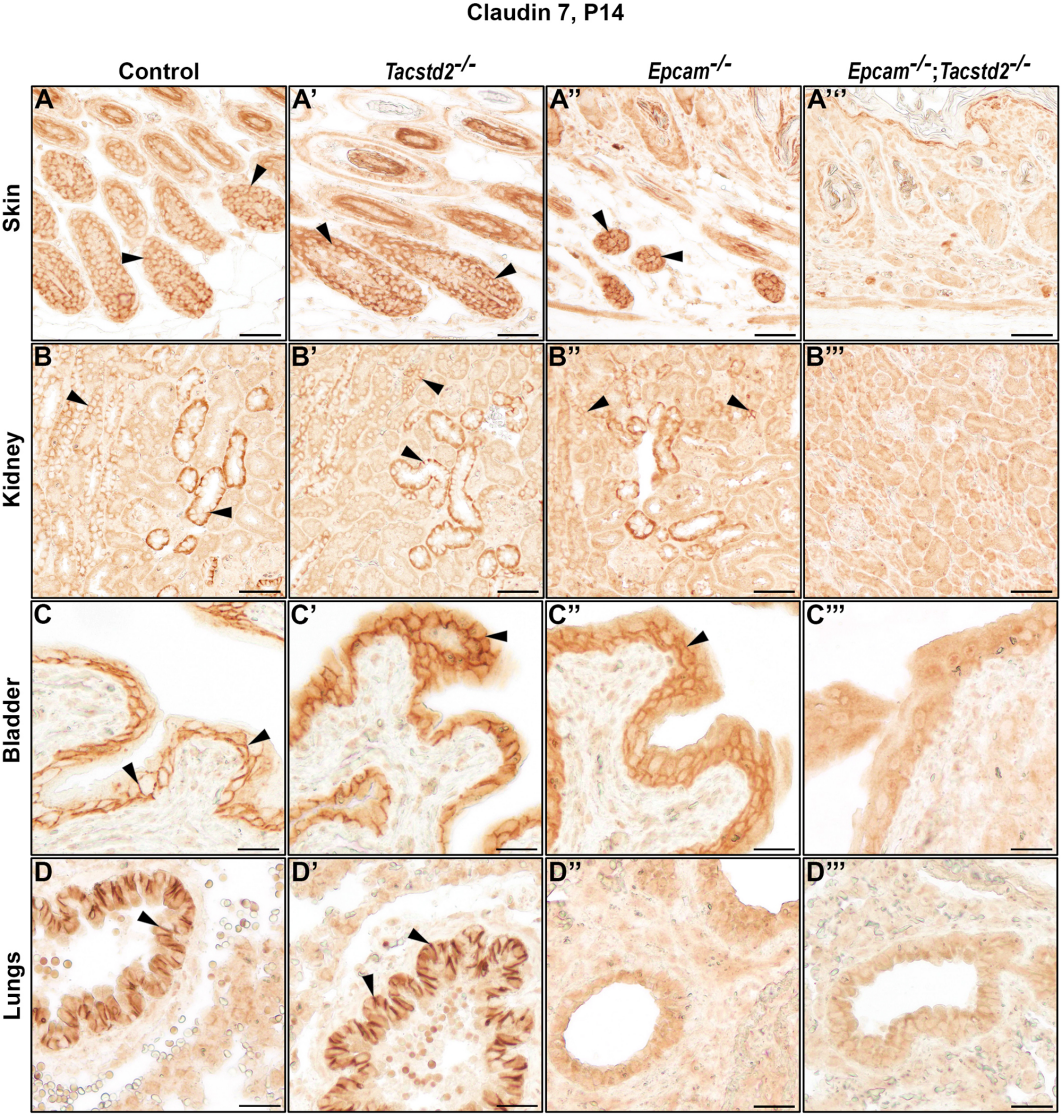
**Cell surface localization of claudin-7 depends on EPCAM and TROP2.** (A-D‴) Immunohistochemical analysis of claudin-7 protein expression in hair follicles in skin, kidney, bladder and lungs from 2-week-old control (A-D), *Tacstd2^−/−^* (A′-D′), *Epcam^−/−^* (A″-D″) and *Epcam^−/−^;Tacstd2^−/−^* (A‴-D‴) mice. Cell surface localization of claudin-7 is indicated by arrowheads. Expression of EPCAM or TROP2 is needed to express and localize claudin-7 to cell surface. Scale bars: A-B‴, 50 μm; C-D’’’, 25 μm.

By immunohistochemistry, cell surface-associated claudin-7 was detected in many epithelia expressing EPCAM and/or TROP2, including those of small and large intestines, bile ducts, exo- and endocrine pancreas, hair follicles, acini and ducts in salivary gland, collecting ducts in kidney, bladder, and airways, but not in epithelia of tongue, forestomach, interfollicular epidermis, thymus, pancreatic ducts, kidney glomeruli and proximal tubules, lung, or cornea (Figs 6A-E, 7A-D, arrowheads, summarized in Table 1). No claudin-7 signal was observed outside the epithelium in any of the organs. Whereas none of the tissues from TROP2-deficient mice showed any impact on level or cellular localization of claudin-7 protein (Figs 6A'-E′, 7A′-D′), inactivation of EPCAM led to a loss of membrane-associated claudin-7 in all epithelia that did not express TROP2, including the intestines, bile ducts, pancreas and salivary gland acini (Fig. 6A″-E″). In contrast, expression and membrane localization of claudin-7 was largely or completely retained in EPCAM-deficient tissues expressing TROP2, such as hair follicles, salivary gland ducts, kidneys, and bladder (Figs 6E*″*, 7A″-C″) but was lost when both EPCAM and TROP2 were missing (Figs 6E‴,7A‴-C‴). The only exception was airway epithelium, in which no membrane-associated claudin-7 was detected in EPCAM single-deficient mice, despite detectable expression of TROP2 (Fig. 7D″, see also Fig. 1I′).

**Table 1.**
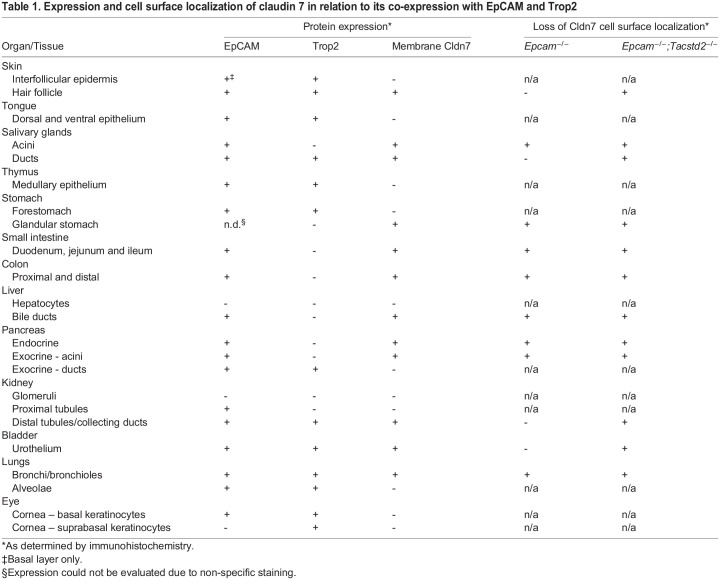
Expression and cell surface localization of claudin 7 in relation to its co-expression with EpCAM and Trop2

## DISCUSSION

EPCAM and its only mammalian homolog TROP2 are cell surface proteins widely expressed in mouse and human epithelia (Momburg et al., 1987; Stepan et al., 2011). Widespread expression of EPCAM and TROP2 appears to be at odds with the very tissue-specific effect of the loss of function of either of the two proteins on mouse and human development, typically attributed to a near-complete co-localization and a possible functional compensation between EPCAM and TROP2 (Wang et al., 2011; Schnell et al., 2013; Trerotola et al., 2013; Wu et al., 2017). In this study, we have performed a direct comparison of the expression of the two homologs in developing mouse tissues and generated mice lacking either or both proteins to investigate their unique and shared functions.

Western blot and immunohistological analysis showed that although the two homologs are indeed expressed in most developing tissues, they frequently do not localize to the same epithelial compartments. In stratified epithelia, including those of the skin, oral cavity and cornea, expression of EPCAM is typically confined to less differentiated, basal layers, whereas TROP2 was detected throughout both basal and differentiated keratinocytes (Fig. 1; Fig. S1). At the same time, a number of simple epithelia, such as those of small and large intestines, exo- and endocrine pancreas, bile ducts, serous and mucous acinar cells in salivary glands, or proximal tubules in kidney, only express EPCAM but not TROP2. This would indicate that in a significant subpopulation of epithelial cells, functional compensation between the two homologs is not likely due to a lack of coordinated expression. Indeed, in addition to previously reported CTE-like defects in small and large intestines, we show that mice lacking EPCAM present with hair follicle hypoplasia leading to near complete alopecia, moderate-to-severe hyperkeratinization in skin and forestomach, glomerular hypoplasia and kidney malfunction indicated by severe proteinuria, as well as abnormal production and/or secretion of pancreatic digestive enzymes, suggesting that the critical role of EPCAM goes well beyond development of intestinal tissues. Furthermore, it should be noted that we limited our evaluation of the impact of EPCAM and TROP2 deficiency to gross macroscopic and histological analysis of major tissues. Therefore, it is plausible that the loss of EPCAM may lead to additional, more subtle defects that do not manifest as changes in overall tissue architecture.

Our findings contrast with previous reports that limit the impact of EPCAM-deficiency in mice to development of intestinal tissues. The apparent difference in observed phenotypes is most likely a result of a significantly longer lifespan of EPCAM-deficient mice used in this study. Compared to previously reported mouse models that were all generated in a mixed 129/C57 background and typically did not survive beyond 5-7 days after birth, our CRISPR-mediated *Epcam* gene inactivation was performed in FVB/N background and resulted in an average lifespan of about 3 weeks, thus enabling the unmasking of additional developmental abnormalities not obvious at earlier time points. Indeed, small and large intestines were the only tissues visibly affected by loss of EPCAM immediately after birth in our study. Alternatively, the apparent discrepancy may also result from the nature of the mutation in the *Epcam* gene. Unlike our current model, which relies on a frameshift and premature termination to preclude any expression of functional EPCAM protein, several of the previous EPCAM-deficient mouse models have been generated by gene trapping - a technique that has been shown at times to lead to an incomplete gene inactivation (List et al., 2007; Guerra et al., 2012; Lei et al., 2012) or by an in-frame deletion of exon 4 of *Epcam* gene in order to mimic a mutation often found in human CTE patients (Mueller et al., 2014). Although not likely, it is possible that in principle these modifications may lead to, respectively, a low-level expression of wildtype EPCAM, and a fully or a partially functional truncated EPCAM protein that might prevent the manifestation of defects in extraintestinal tissues. With respect to human studies, it should be noted that several of the epithelia that exhibit EPCAM-specific expression in mice, including pancreas or acinar cells within the salivary glands, have previously been shown to express TROP2 in humans. Thus, a more expansive expression of TROP2 in human tissues may provide a potential explanation for more tissue-restrictive impact of EPCAM deficiency in CTE patients (Stepan et al., 2011).

In contrast to EPCAM, lack of TROP2 did not have an appreciable impact on mouse development, health and survival. Although this may indicate that TROP2 does not play an essential role in mouse, we show that TROP2 is rarely expressed in cells that do not also express EPCAM, which then may be available to compensate for the loss of its homolog, thus explaining the apparent expendability. Indeed, the only reported effects of TROP2 deficiency on mouse or human development are light-induced corneal opacity and gelatinous drop-like corneal dystrophy, respectively, as well as an increased epidermal permeability identified in our current study (Fig. 4) (Nakatsukasa et al., 2010; Nagahara et al., 2020). In both cornea and in epidermis, these defects are expected to be a result of impaired epithelial barrier function that resides in differentiated layers of their corresponding epithelia, neither of which expresses detectable levels of EPCAM (Fig. 1).

Our data are consistent with EPCAM and TROP2 having overlapping biological and molecular function. First, inactivation of TROP2 increased severity of developmental defects in EPCAM-deficient mice in epithelia that normally express both proteins, including skin and hair follicles, forestomach and kidney, and further limited postnatal growth and survival. This was in contrast to tissues that only expressed EPCAM, such as small and large intestines, or pancreas, where loss of TROP2 did not appear to further aggravate the defects resulting from the EPCAM deficiency. These findings strongly indicate an ability of TROP2 to at least partially compensate for the loss of EPCAM function. Second, the two proteins were largely interchangeable in their ability to maintain proper expression and localization of claudin 7, as indicated by loss of cell surface-associated claudin-7 only in cell that lack both EPCAM and TROP2 (Figs 6 and 7). However, the fact that on histological and macroscopic level tissues that express both proteins, such as skin, stomach, or kidney, were affected by loss of EPCAM, but not by loss of TROP2, suggests that TROP2 may not be able to fully compensate for the loss of its homolog, and therefore, may not be functionally equivalent to EPCAM. This is consistent with a recent report by Nakato et al., showing that ectopic expression of TROP2 in intestinal epithelium was able to partially, but not completely, ameliorate CTE-like defects in mice (Nakato et al., 2020). Further studies are needed to address possible reasons for this incomplete redundancy that may include differing affinities to individual downstream targets, such as claudin-7 or claudin-1, incomplete subcellular co-localization, or tissue-to-tissue variation in the ratio of TROP2 and EPCAM expression and/or stability.

It is well established that both EPCAM and TROP2 have the ability to bind and stabilize at least some of the claudins, especially claudin-7, on the cell surface (Nakatsukasa et al., 2010; Wu et al., 2013). In addition, claudin-7-deficient mice exhibit severe defects in intestinal development similar to those found in EPCAM-deficient mice (Ding et al., 2012). We have similarly observed strong reduction in expression and a loss of membrane localization of claudin-7 in the intestines and pancreas from mice that lack EPCAM (Figs 5-7). However, several other tissues affected by EPCAM or TROP2 deficiency either did not express membrane-associated claudin-7 (forestomach, interfollicular epidermis), or did not present with a substantial change in claudin-7 expression and/or subcellular distribution in the absence of EPCAM (hair follicles, kidney). At the same time, loss of claudin-7 expression in liver bile ducts or salivary gland acinar cells was not associated with any obvious developmental abnormality. Although the latter does not preclude a possibility of more subtle defects that do not manifest at the level of tissue architecture, taken together, our data does not provide a convincing evidence for a uniform link between EPCAM and claudin-7 function, at least in the extraintestinal tissues. Whether this is an indication of interaction with additional downstream targets, including other claudins, and possibly in a tissue-specific manner, remains to be determined.

In conclusion, we demonstrate that in mice the critical role of EPCAM is not limited to intestinal tissues and affects a significant subpopulation of developing epithelia, and that although loss of TROP2 does not have an obvious impact on overall mouse development and survival, it does exacerbate many of the developmental defects caused by EPCAM deficiency in tissues that normally express both proteins. We have also shown that TROP2 is able to partially compensate for the loss of EPCAM in stabilizing claudin-7 expression and localization. These findings identify EPCAM and TROP2 as regulators of epithelial development in both intestinal and extraintestinal tissues. It may be worthwhile to assess whether any of these defects also present in CTE patients and possibly contribute to the disease burden.

## MATERIALS AND METHODS

### Generation of Epcam- and Tacstd2-deficient mouse strains

All experiments involving mice were performed in an Association for Assessment and Accreditation of Laboratory Animal Care International-accredited vivarium following Institutional Guidelines and Standard Operating Procedures as approved by the NIDCR Institutional Animal Care and Use Committee.

To generate *Epcam*- and *Tacstd2*-deficient mouse strains, two guide RNAs targeting the exon 4 of *Epcam* gene or a 5′ region of *Tacstd2* gene (Table S1 for guide sequences) were designed using the CHOP-CHOP CRISPR guide RNA design tool (http://chopchop.cbu.uib.no/, (Labun et al., 2019) and synthesized by Horizon Discovery Biosciences (Cambridge, UK). 50 ng/µl guide RNA and 100 ng/µl recombinant *Sp*Cas9 protein (PNA Bio, Newbury Park, CA, USA) were pre-mixed in 10 mm Tris/HCl, pH 7.4, 0.1 mm EDTA, and microinjected into the male pronucleus of FVB/NJ zygotes, followed by implantation into pseudopregnant FVB/NJ female mice. All founders were screened for changes in the targeted regions by PCR amplification followed by DNA sequencing (Table S1 for primer sequences, 35 cycles at 94°C/56°C/72°C, 1 min per step). Mice carrying gene-inactivating mutations were bred to FVB/NJ wildtype mice to test the germ-line transmission and to establish stable mouse lines. One line carrying a predicted null mutation per gene was further bred to generate experimental animals. Genotyping was performed on ear or tail clips from newborn to two-week-old mice using primers and conditions indicated above. All experiments were littermate controlled.

### Tissue extraction and histological analysis

Mice were euthanized on postnatal days 1, 14, or 20, tissues were dissected and immediately fixed in aqueous-buffered zinc formalin fixative (Z-Fix, Anatech Ltd., Battle Creek, MI, USA) for 24 h at room temperature prior to paraffin embedding and sectioning (Histoserv, Germantown, MD, USA). Newborn mice were sectioned sagittally. 5 µm thick sections were stained with hematoxylin and eosin (H&E) and Alcian blue/PAS (both performed by Histoserv) or used for immunohistochemistry as described below.

### Immunohistochemistry

Five µm thick sections from formalin-fixed, paraffin-embedded mouse tissues were immunostained after antigen retrieval by incubation for 20 min at 100°C in 0.01 M sodium citrate buffer, pH 6.0 essentially as described previously (Szabo et al., 2016). Briefly, the sections were blocked with 2.5% bovine serum albumin (Fraction V, MP Biomedicals, Solon, OH, USA) in PBS and incubated overnight at 4°C with goat anti-hEPCAM (R&D Systems, Minneapolis, MN, USA), rabbit anti-hTROP2 (ab214488, Abcam, Cambridge, MA, USA), or rabbit anti-hClaudin-7 (Life Technologies, Rockford, IL, USA) primary antibodies (see Table S2 for further information on antibodies). Bound antibodies were visualized using biotin-conjugated anti-rabbit, or anti-goat secondary antibodies (Vector Laboratories, Burlingame, CA, USA) and a Vectastain ABC Kit (Vector Laboratories), using 3,3′-diaminobenzidine as the substrate (Sigma-Aldrich, St. Louis, MO, USA). All microscopic images were acquired on Olympus BX43 microscope using Olympus DP74 digital camera with cellSens Entry system (all Olympus, Melville, NY, USA).

### Protein extraction and Western blot analysis

Mouse tissues were collected at postnatal day 14 or 21, snap-frozen in liquid nitrogen, and stored at −80°C until further use. Western blot analysis was performed essentially as described elsewhere (Szabo et al., 2019). Briefly, the tissues were homogenized in 2% SDS and 10% glycerol in 62.5 mM Tris/Cl pH 6.8 containing Protease Inhibitor Cocktail (Sigma-Aldrich). The lysates were cleared by centrifugation at 16,000 ***g*** for 10 min at 4°C to remove the tissue debris and the protein concentration in supernatant was determined by BCA assay (Pierce, Rockford, IL, USA). 60 µg of total protein was mixed with 4×SDS sample buffer (NuPAGE, Invitrogen, Carlsbad, CA, USA) containing 1 M β-mercaptoethanol (Sigma-Aldrich), boiled for 5 min at 99°C, and run on 4–12% BisTris NuPAGE gels using 1xMOPS running buffer (both Invitrogen). Separated proteins were transferred to PVDF membranes (0.2 μm, Invitrogen) and blocked for 30 min at room temperature with 5% nonfat dry milk in Tris-buffered saline, 0.05% Tween 20 (TBS-T). Membranes were incubated with primary antibody overnight at 4°C, followed by incubation with alkaline phosphatase-conjugated secondary antibody for 1.5 h at room temperature (see Table S2 for further information on antibodies). Alkaline phosphatase activity was visualized using nitro-blue tetrazolium and 5-bromo-4-chloro-3'-indolylphosphate substrates (Sigma-Aldrich). Data shown are representative of at least two independent Western blot experiments. When indicated, intensity of Western blot signal was quantified using Adobe Photoshop 2022.

### Transepidermal water loss assay

The assay was performed essentially as described previously (List et al., 2007). Newborn pups were separated from their parents to prevent fluid intake and placed in a 37°C incubator. The rate of transepidermal water loss was measured as the reduction of body weight once an hour over a period of 6 h.

### Urine Albumin and Creatinine assay

To collect urine, 2-3 week old mice were held over plastic sheet for up to 30 s with lightly stroking the belly to allow urination. Urine was then transferred into an Eppendorf tube, cleared by centrifugation, and stored at −80°C until analyzed. 1 µl of urine was used to measure albumin concentration by colorimetric ELISA assay (Mouse Albumin ELISA kit, ab108792, Abcam) according to the manufacturer's protocol. To determine creatinine concentration, 2 µl of urine were used in a colorimetric assay (Creatinine Assay Kit, ab65340, Abcam). At the conclusion of the assay, absorbances were read on Synergy Neo microplate reader (Agilent Biotek, Santa Clara, CA, USA). All standards and samples were measured in duplicates and the concentrations were determined from the average of the two values.

### Statistical analysis

Allele distribution at birth was evaluated using chi-square analysis of the observed versus the expected numbers of *Epcam^+/+^*, *Epcam^+/−^* and *Epcam^−/−^* mice among *Tacstd2^+/+^*, *Tacstd2^+/−^* and *Tacstd2^−/−^* offspring from *Epcam^+/−^;Tacstd2^+/−^* parents.

Postnatal survival was analyzed using Mantel-Cox log-rank test. Median survival was analyzed using one-way ANOVA. Postnatal growth of control, *Epcam^−/−^*, and *Tacstd2^−/−^* single-deficient mice was analyzed by comparing linear regression slopes of the average total body weights of mice of different genotypes measured daily. Comparison of postnatal growth of *Epcam^−/−^* single-deficient and *Epcam^−/−^;Tacstd2^−/−^* double-deficient mice was analyzed by two sample Student's *t*-test, two tailed of the total body weights at each time point.

Transepidermal fluid loss was analyzed by comparing linear regression slopes of the average total body weights of newborn mice of different genotypes measured once an hour for 6 h.

Albumin and creatinine content in urine was measured in duplicates and the individual concentrations as well as the albumin-to-creatinine ratios (ACR) were determined from the average of the two values. Results were statistically evaluated using a one-way ANOVA.

All statistical analysis was performed using GraphPad Prism software (GraphPad Prism ver.8.4.1, GraphPad Software, Inc., La Jolla, CA, USA)

## Supplementary Material

Supplementary information
