# Supplementary material for: EPCAM and TROP2 share a role in claudin stabilization and development of intestinal and extraintestinal epithelia in mice
Source: Biol Open. 2022 Jul 11;11(7):bio059403. doi: 10.1242/bio.059403 (PMC9294608; doi:10.1242/bio.059403)
Supplement: Supplementary information [file biolopen-11-059403-s1.pdf]

Supplementary figure 1.

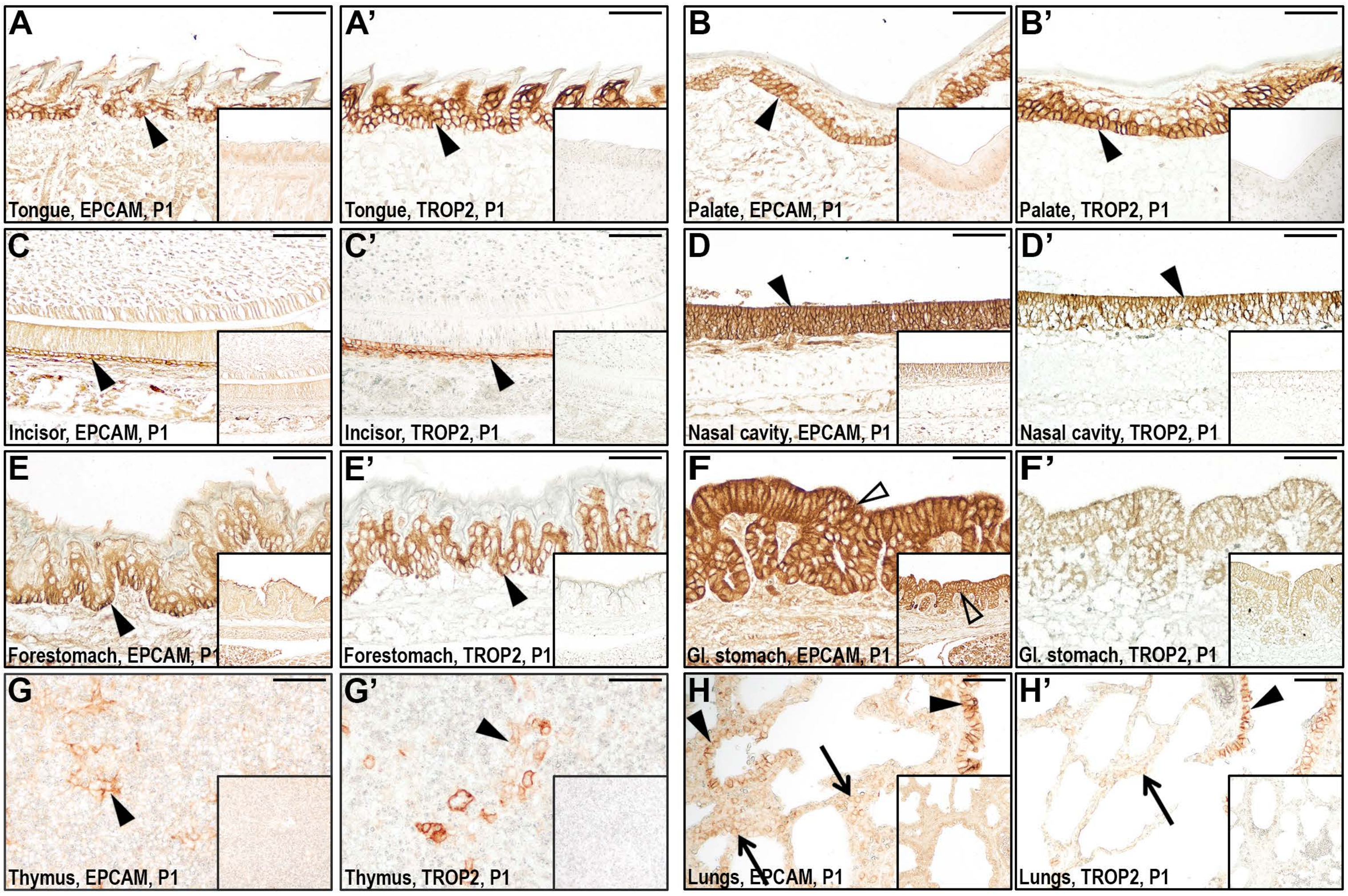

Fig. S1. EPCAM and TROP2 are co-expressed in developing mouse epithelia. (A-H'). Immunohistochemical analysis of EPCAM (**A-H**) and TROP2 (**A'-H'**) protein expression in tongue (**A,A'**), palate (**B,B'**), incisors (**C,C'**), nasal cavity (**D,D'**), forestomach (**E,E'**), glandular stomach (**F,F'**), thymus (**G,G'**), and lungs (**H,H'**) from newborn wildtype mice. Insets show corresponding tissues from EPCAM-deficient or TROP2-deficient mice used as controls for antibody specificity. Expression of EPCAM and TROP2 is indicated by arrowheads. Co-expression of EPCAM and TROP2 was detected in stratified epithelia within oral cavity and forestomach (**A-B', E,E'**), pseudostratified epithelium in nasal cavity (**D,D'**), as well as in epithelial cells of stratum intermedium in developing tooth (**C,C'**), thymus medulla (**G,G'**), bronchiole (**H,H'**, arrowheads) and alveolae (**H,H'**, arrows). EPCAM expression in glandular stomach could not be evaluated due to apparent non-specific staining (**F,F'**, open arrowheads). Size bars: (**A-B', E-G'**) 50 µm, (**C-D'**) 75 µm.

Supplementary figure 2.

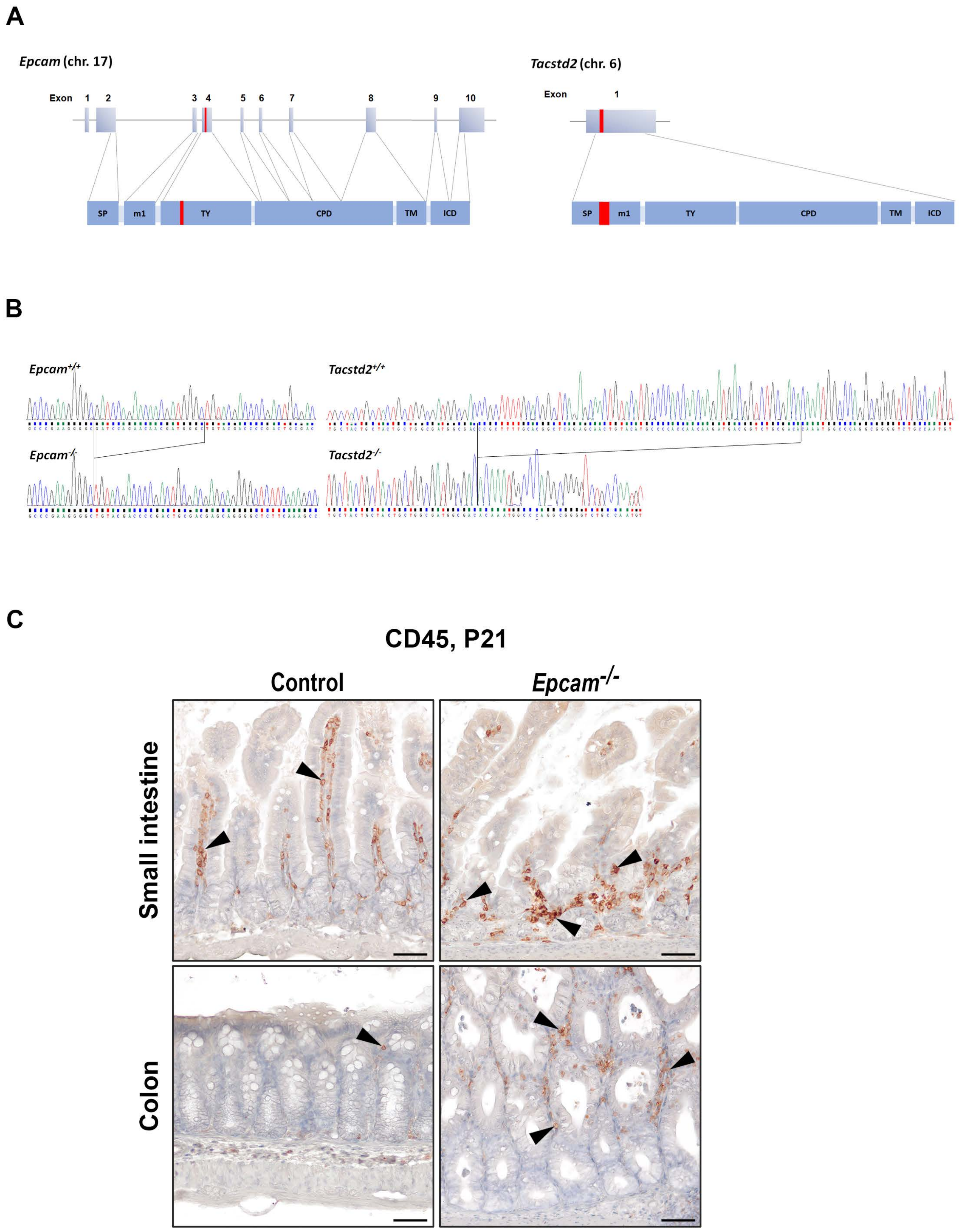

Fig. S2. Loss of EPCAM triggers intestinal inflammation. (**A**). Schematics of CRISPR/Cas9-mediated targeting leading to a 20 bp deletion within exon 4 of mouse *Epcam* (left), and a 61 bp deletion within the single-exon *Tacstd2* (right) gene. The deleted parts of the open reading frames and the corresponding regions in the encoding proteins are indicated in red. Only the portion of the protein N-terminal to the mutation site can be correctly translated. Abbreviations in (**A**): SP, signal peptide; m1, EPCAM motif 1/cysteine rich domain; TY, thyroglobulin 1A domain; CPD, cysteine poor domain; TM, transmembrane domain; ICD, intracellular domain. (**B**). Representative chromatogram results of DNA sequencing of wildtype (top) and (bottom) *Epcam*$^{-/-}$ (left) and *Tacstd2*$^{-/-}$ (right) mice, confirming successful targeting. **(C).** Immunohistochemical analysis of CD45 protein expression in small intestines (top) and colon (bottom) from 21 days old control (left) and *Epcam*$^{-/-}$ (right) mice. Examples of CD45-positive cells are indicated by arrowheads. Size bars: 50 µm.

Biology Open • Supplementary information

Supplementary figure 3.

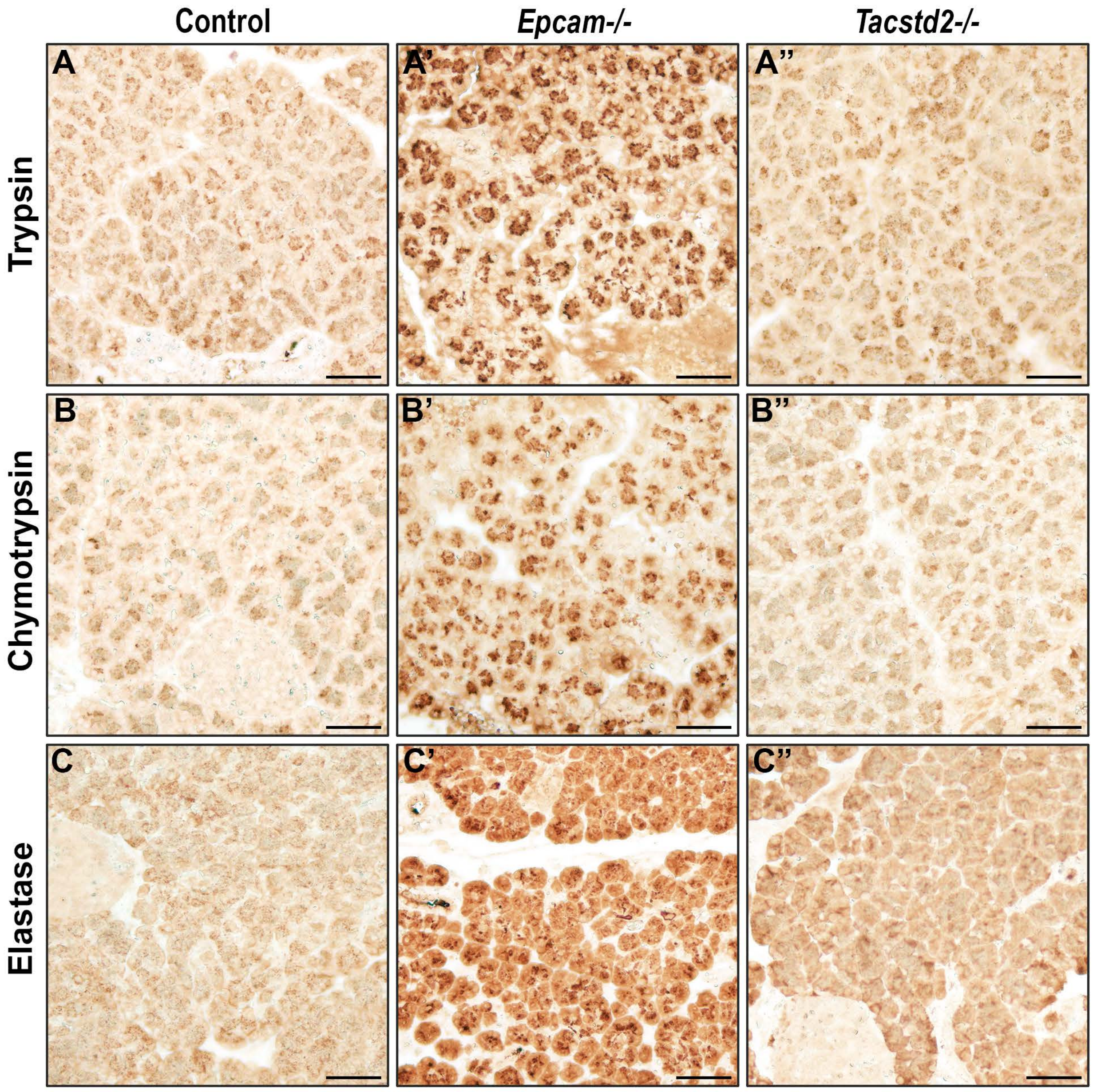

Fig. S3. Loss of EPCAM affects production of pancreatic enzymes. Immunohistochemical detection of trypsin (**A-A''**), chymotrypsin (**B-B''**), and pancreatic elastase **(C-C'')** in pancreas from 21 days old control **(A-C),** *Epcam*$^{-/-}$ **(A'-C')**, and *Tacstd2*$^{-/-}$ **(A''-C'')** mice. EPCAM-deficiency leads to an increased intracellular content of digestive enzymes in exocrine pancreas. Size bars: 50 µm.

Supplementary figure 4.

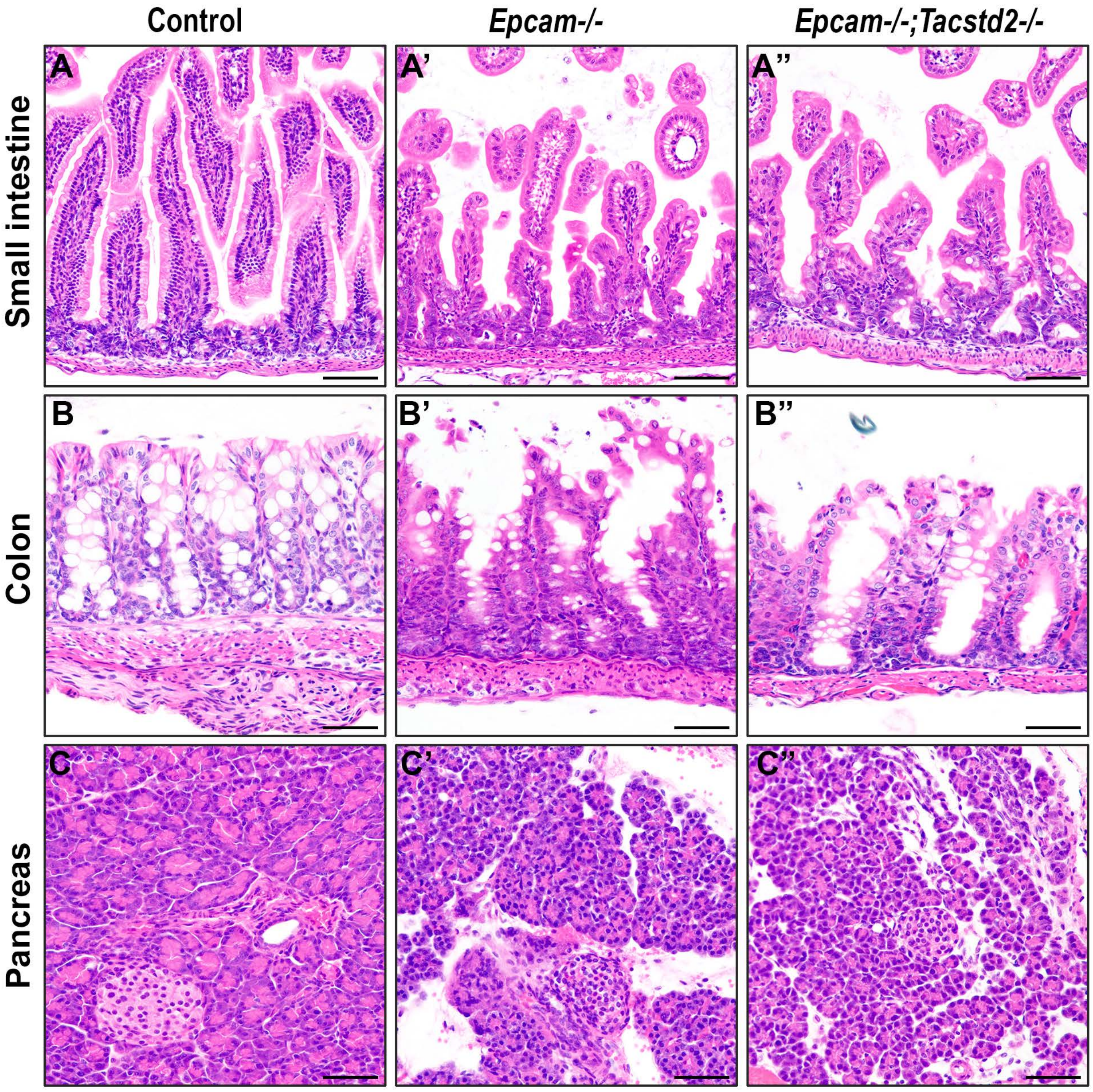

Fig. S4. Loss of TROP2 does not affect development of intestines and pancreas in EPCAM-deficient mice. (**A-C"**). H&E stain of small intestines (jejunum, **A-A"**), colon (**B-B"**) and pancreas (**C-C"**) from 14 days old control (**A-C**), and their littermate *Epcam*$^{-/-}$ (**A'-C'**) and *Epcam*$^{-/-}$*;Tacstd2*$^{-/-}$ (**A"-C"**) mice. Defects resulting from the loss of EPCAM in these tissues were not further affected by the absence of TROP2. Size bars: (**A-C"**) 75 µm.

Biology Open • Supplementary information

Supplementary figure 5.

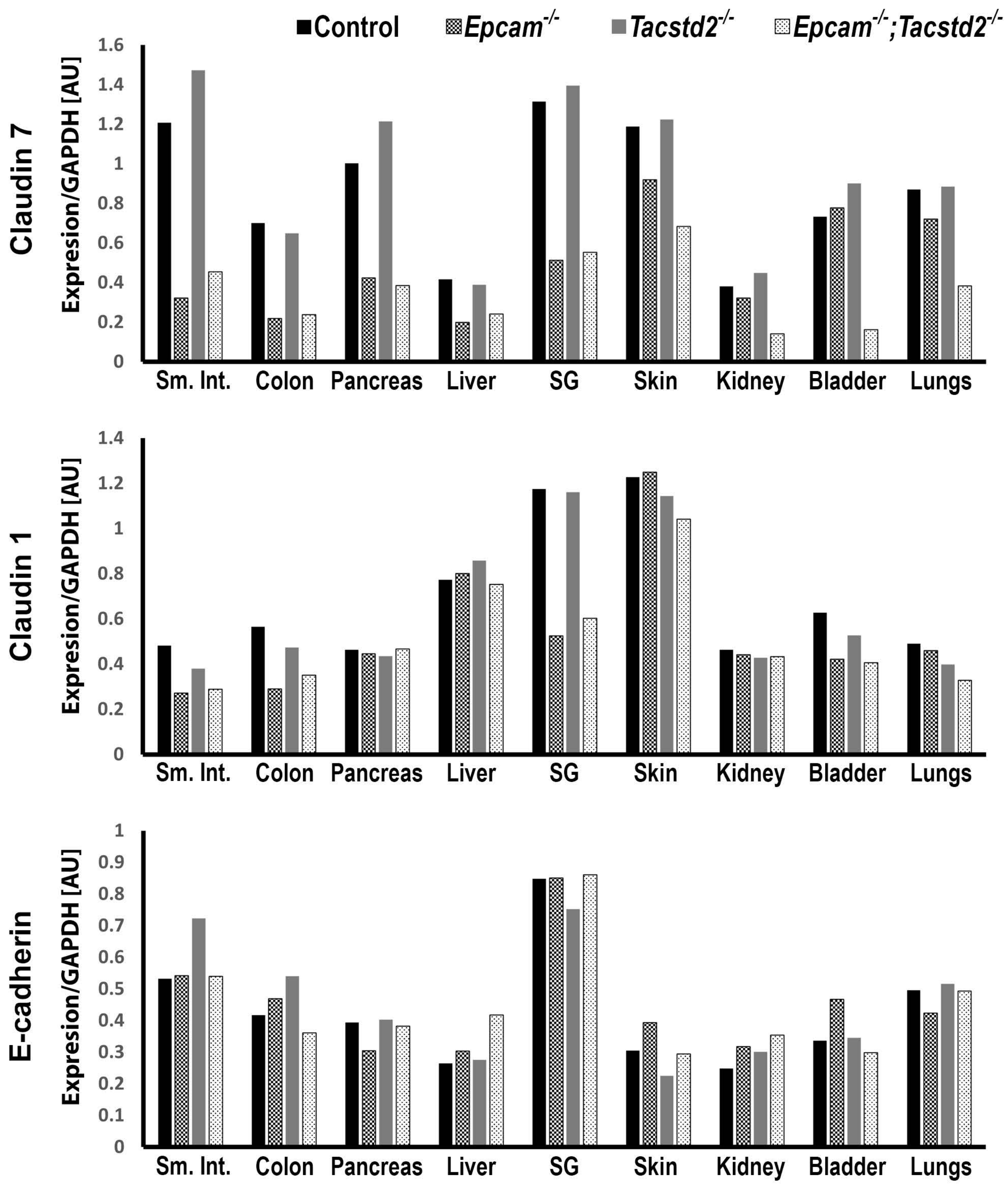

Fig. S5. Expression of epithelial markers in mice lacking EPCAM and/or TROP2. Quantification of Western blot signal shown in Figure 5, representing expression of claudin-7 (top), claudin-1 (middle), and E-cadherin (bottom) in tissues from 14 days old wildtype (Control), *Epcam*$^{-/-}$, *Tacstd* $^{-/-}$, and *Epcam*$^{-/-}$;*Tacstd2*$^{-/-}$ double-deficient mice. Each bar represents average value from two mice per genotype and tissue, relative to GAPDH (see Figure 5).

Biology Open • Supplementary information

Table S1. Sequences of guide RNAs and genotyping primers used in this study.

Gene	Guide target/Primer sequences
Gene targeting	
Epcam	Guide 1: 5'-gaggaggataaagcccgaag-3'<br>Guide 2: 5'-tgactcacagcaagtctggg-3'
Tacstd2	Guide 1: 5'-gatggcgacccgcttttgca-3'<br>Guide 2: 5'-gtcgcagaccgtcatcttgt-3'
Screening and genotyping	
Epcam	5'-aatccttggcctggatggc-3'<br>5'-ggaaagctcactcactagg-3'
Tacstd2	5'-tctggtctgtagttggagg-3'<br>5'-agcagcaggcacttggaag-3'

Table S2. List of antibodies used in the study

**Immunohistochemistry**			
**Primary**			
**Antigen**	**Manufacturer**	**Cat. No.**	**Concentration**
EpCAM	R&D Systems, Minneapolis, MN	AF960	5 ug/ml
Claudin-7	Life Technologies, Rockford, IL	34-9100	2.5 ug/ml
Trop2	Abcam, Cambridge, MA	ab214488	5 ug/ml
CD45	Abcam, Cambridge, MA	ab23910	2.5 ug/ml
Trypsin	R&D Systems, Minneapolis, MN	AF3848	5 ug/ml
Chymotrypsin	R&D Systems, Minneapolis, MN	AF6907	5 ug/ml
Elastase	Invitrogen	PA5-28155	5 ug/ml
**Secondary**			
Anti-rabbit	Vector Laboratories, Burlingame, CA	BA-1000	2.5 ug/ml
Anti-goat	Vector Laboratories, Burlingame, CA	BA-9500	2.5 ug/ml
Anti-rat	Vector Laboratories, Burlingame, CA	BA-9400	2.5 ug/ml
Anti-sheep	Vector Laboratories, Burlingame, CA	BA-6000	2.5 ug/ml
**Western Blot**			
**Primary**			
EpCAM	R&D Systems, Minneapolis, MN	AF960	1 ug/ml
Claudin-7	Life Technologies, Rockford, IL	34-9100	0.5 ug/ml
Trop2	Abcam, Cambridge, MA	ab214488	1 ug/ml
GAPDH	Abcam, Cambridge, MA	ab9485	0.5 ug/ml
**Secondary**			
Anti-rabbit	Dako Cytomation, Carpinteria, CA	D0487	1 ug/ml
Anti-goat	Sigma, St.Louis, MO	A4187	1 ug/ml

Biology Open • Supplementary information
